# Parental inflammatory bowel disease and autism in children

**DOI:** 10.1038/s41591-022-01845-9

**Published:** 2022-06-02

**Authors:** Aws Sadik, Christina Dardani, Panagiota Pagoni, Alexandra Havdahl, Evie Stergiakouli, The iPSYCH Autism Spectrum Disorder Working Group, Jakob Grove, Golam M. Khandaker, Sarah A. Sullivan, Stan Zammit, Hannah J. Jones, George Davey Smith, Christina Dalman, Håkan Karlsson, Renee M. Gardner, Dheeraj Rai

**Affiliations:** 1 Population Health Sciences, Bristol Medical School, University of Bristol, Bristol, UK; 2 Avon and Wiltshire Partnership NHS Mental Health Trust, Bath, UK; 3 Medical Research Council Integrative Epidemiology Unit, Bristol Medical School, University of Bristol, Bristol, UK; 4 Department of Mental Disorders, Norwegian Institute of Public Health, Oslo, Norway; 5 Nic Waals Institute, Lovisenberg Diakonale Hospital, Oslo, Norway; 6 PROMENTA Research Center, Department of Psychology, University of Oslo, Oslo, Norway; 7 Department of Psychiatry, University of Cambridge, Cambridge, UK; 8 Cambridgeshire and Peterborough NHS Foundation Trust, Cambridge, UK; 9 National Institute of Health and Care Research Biomedical Research Centre, University Hospitals Bristol and Weston NHS Foundation Trust and University of Bristol, Bristol, UK; 10 Division of Psychological Medicine and Clinical Neurosciences, Cardiff University, Cardiff, UK; 11 Department of Global Public Health, Karolinska Institutet, Stockholm, Sweden; 12 Centre for Epidemiology and Community Medicine, Stockholm County Council, Stockholm, Sweden; 13 Department of Neuroscience, Karolinska Institutet, Stockholm, Sweden; 14 The Lundbeck Foundation Initiative for Integrative Psychiatric Research, iPSYCH, Aarhus, Denmark; 15 Department of Biomedicine and Center for Integrative Sequencing, iSEQ, Aarhus University, Aarhus, Denmark; 16 Center for Genomics and Personalized Medicine, CGPM, Aarhus University, Aarhus, Denmark; 17 Bioinformatics Research Center, Aarhus University, Aarhus, Denmark

**Keywords:** Risk factors, Epidemiology

## Abstract

Evidence linking parental inflammatory bowel disease (IBD) with autism in children is inconclusive. We conducted four complementary studies to investigate associations between parental IBD and autism in children, and elucidated their underlying etiology. Conducting a nationwide population-based cohort study using Swedish registers, we found evidence of associations between parental diagnoses of IBD and autism in children. Polygenic risk score analyses of the Avon Longitudinal Study of Parents and Children suggested associations between maternal genetic liability to IBD and autistic traits in children. Two-sample Mendelian randomization analyses provided evidence of a potential causal effect of genetic liability to IBD, especially ulcerative colitis, on autism. Linkage disequilibrium score regression did not indicate a genetic correlation between IBD and autism. Triangulating evidence from these four complementary approaches, we found evidence of a potential causal link between parental, particularly maternal, IBD and autism in children. Perinatal immune dysregulation, micronutrient malabsorption and anemia may be implicated.

## Abstract (web summary)

Evidence from four complementary methodological approaches, harnessing genotype and phenotype data, identifies potential causal links between inflammatory bowel disease in parents and autism in children.

## Main

Autism spectrum disorder (autism) is a chronic neurodevelopmental condition with a highly variable clinical manifestation^1^. Beyond the core phenotypic expressions of autism (social communication difficulties and restricted interests/repetitive behaviors), emerging evidence suggests that almost half of autistic individuals present with gastrointestinal symptoms (median prevalence 47%, in a review of studies published between 1980 and 2017^2^). In addition, a recent study of 48,762 autistic children and 243,810 controls in the United States (US), suggested that children with autism were approximately 47% more likely to be diagnosed with Crohn’s disease (Crohn’s) and 94% more likely to be diagnosed with ulcerative colitis (UC) compared with controls^3^.

Crohn’s and UC are the major subtypes of inflammatory bowel disease (IBD), a chronic condition associated with immune system dysregulation, intestinal microbiome alterations, micronutrient malabsorption and anemia^4–6^. There is evidence suggesting that these characteristics of IBD might be perinatal factors associated with autism^7–10^. On this basis, a potential link between parental IBD and autism in children could be hypothesized. Evidence so far is inconclusive, with only one out of the four registry-based studies in the field^11–13^ indicating an association between maternal UC and autism in children^14^. Moreover, the underlying etiology of any associations is unclear.

**Fig. 1:**
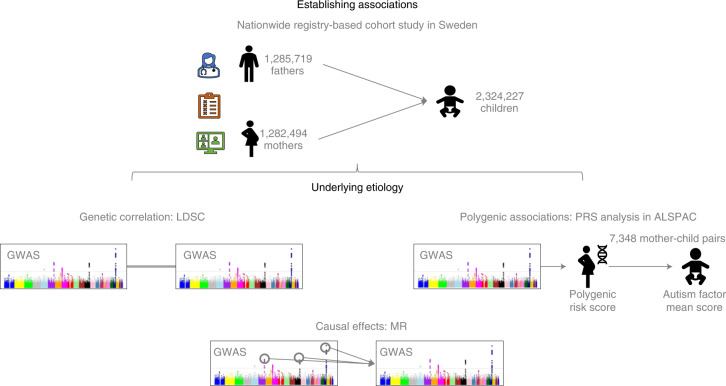
Summary of studies conducted, aiming at investigating the links between parental diagnoses of IBD and offspring autism in children and elucidating their underlying etiology. Manhattan plot icons adapted from a previous publication^57^.

We conducted four complementary studies (Fig. 1 and Table 1) to investigate: (1) associations between parental diagnoses of IBD and autism in children in a nationwide cohort in Sweden; (2) genetic correlation between IBD and autism using genome-wide association study (GWAS) summary statistics; (3) polygenic associations between maternal genetic liability to IBD and autistic traits in children in a large UK birth cohort; and (4) potential causal effects of genetic liability to IBD on autism and the possibility of reverse causation using bidirectional two-sample Mendelian randomization (MR).

**Table 1:** Summary of the research question, data sources used, as well as key strengths and limitations of each methodological approach applied in the present study

Method	Research question	Data sources	Key strengths	Key limitations
**Nationwide registry-based cohort study in Sweden**	Are parental diagnoses of IBD associated with autism in the children?	Medical and administrative registers	Large, diverse total population, intergenerational sample; prospective recording of data; low rate of loss to follow up; large availability of confounder data	Unmeasured confounding; exposure misclassification
**Linkage Disequilibrium score regression**	Is there a shared genetic background between IBD and autism?	GWAS summary data	Use of GWAS summary data instead of twin data or individual-level data maximizes sample sizes and power; indicates genetic correlation due to linkage disequilibrium or pleiotropy	Cannot assess causality
**PRS analysis in the ALSPAC cohort**	Is maternal genetic liability for IBD associated with childhood broad autism phenotype?	GWAS summary data and individual-level genotype and phenotype data	Estimates the underlying genetic liability for IBD, regardless of diagnosis; allows the refinement of the exposure used in the context of an observational study (potentially overcoming exposure misclassification of an observational study); can indicate potentially genetically transmitted versus in-utero effects through the assessment of the maternal versus child’s underlying genetic liability for IBD; large birth cohort; prospectively collected information on the outcome phenotype	Cannot decipher whether the identified associations are causal or instead owing to pleiotropy; polygenic risk scores at lower *P-*value thresholds might not adequately capture the exposure phenotype; attrition can influence association estimates
**Two-sample MR**	Does genetic liability to IBD have a causal effect on autism?	GWAS summary data, exposure proxied by variants robustly associated with the exposure	Using common genetic variants as instruments for IBD allows the assessment of causal effects; allows the assessment of reverse causation; allows the assessment of the influence of pleiotropy	Cannot decipher whether the identified causal effect is of parental origin; can be biased by dynastic effects and assortative mating

## Results

### Study 1: Parental IBD diagnoses and autism in children

Nationwide population-based registers are powerful resources in etiological epidemiology, as they offer large intergenerational samples, prospectively collected data, and minimal loss to follow up and selection bias (Table 1). ‘Psychiatry Sweden’ is a comprehensive national register linkage including individual-level health, demographic, and socioeconomic data across Sweden.

**Extended Data Fig. 1:**
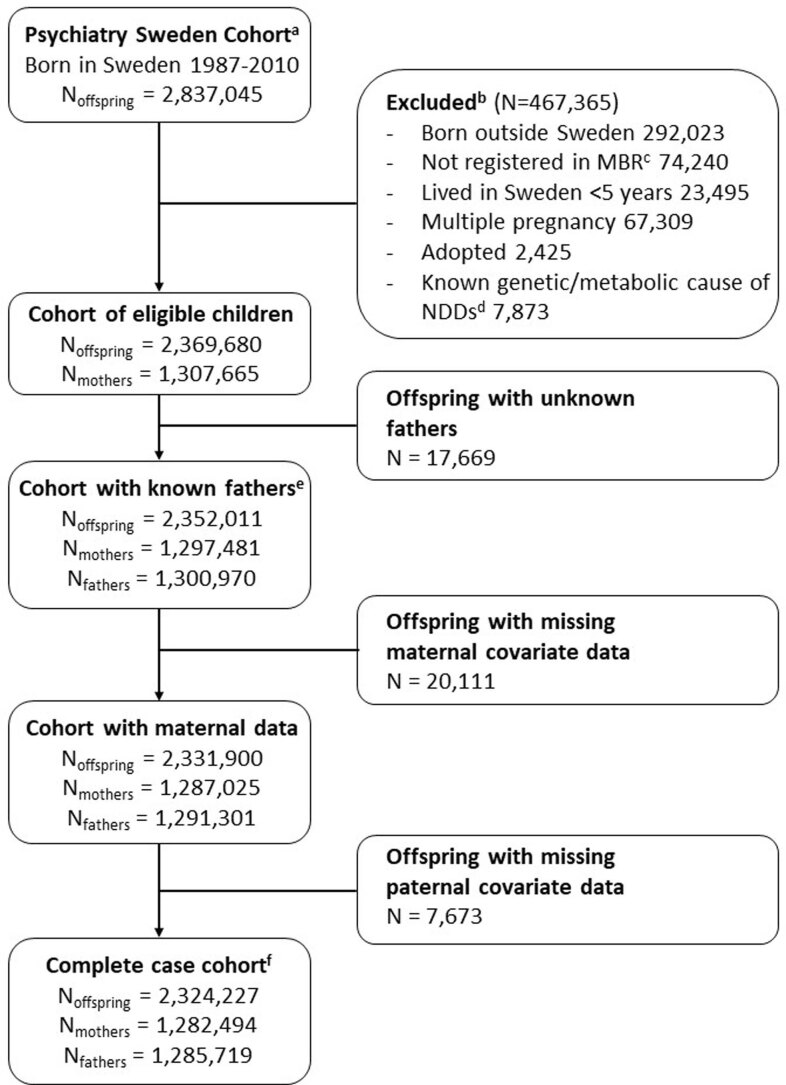
Study sample derivation for the Swedish cohort study. **a,** Children born before 1987 were excluded from the study population. **b,** Individuals were excluded stepwise with the criteria aligned to a previous study (reference ^78^). **c,** Individuals without information from the MBR were excluded. **d,** Children with diagnoses of autism or IDs and genetic or metabolic conditions documented in the MBR that were known to cause neurodevelopmental disorders (NDDs), as the NDDs may be attributable to the condition. **e,** Those whose biological fathers were unknown were excluded. **f,** Those whose biological mothers or fathers lacked data on place of birth, age at delivery, education level, psychiatric history, or family income quintile were excluded.

**Extended Data Fig. 2:**
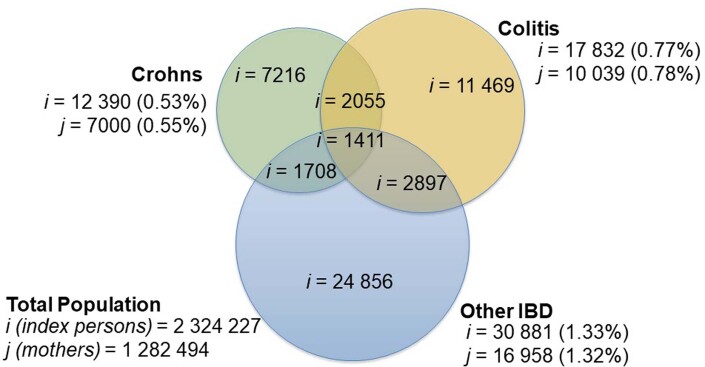
Prevalence and overlap in diagnoses among the mothers included in the present study. Overall prevalences of Crohn’s, UC, and other IBD diagnoses are shown in terms of mothers diagnosed (j) as well as children born to those mothers and followed up for autism in Study 1 (index persons, i). Overlap in the diagnoses is only shown for index persons included in the study, to align with values in Table 2 in the main text, though the extent of overlap is virtually identical if mothers or index persons are displayed. For example, of the 7,000 mothers diagnosed with Crohn’s, 1984 (28.4%) were also diagnosed with colitis, and the analogous proportion of children is 28.0% (3,466/12,390).

**Extended Data Fig. 3:**
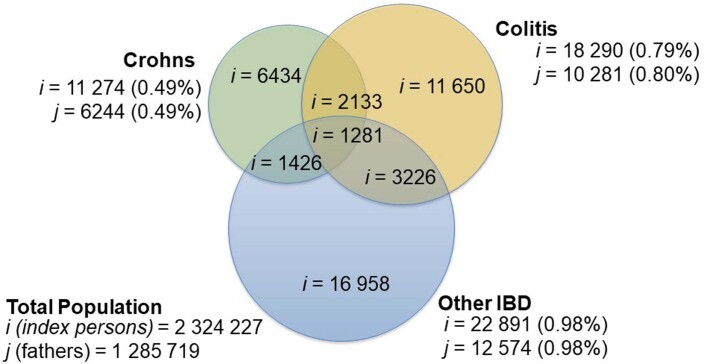
Prevalence and overlap in diagnoses among the fathers included in the present study. Overall prevalences of Crohn’s, UC, and other IBD diagnoses are shown in terms of fathers diagnosed (j) as well as children born to those mothers and followed up for autism in Study 1 (index persons, i). Overlap in the diagnoses is only shown for index persons included in the study, to align with values in Table 2 in the main text, though the extent of overlap is virtually identical if fathers or index persons are displayed. For example, of the 6,244 fathers diagnosed with Crohn’s, 1,925 (30.8%) were also diagnosed with colitis, and the analogous proportion of children is 30.3% (3,414/11,274).

In a sample of 2,324,227 children born to 1,282,494 mothers and 1,285,719 fathers from﻿ ‘Psychiatry Sweden,’ we assessed the associations between parental IBD diagnosis and autism in children (Online Methods, Extended Data Fig. 1, Extended Data Fig. 2, Extended Data Fig. 3, Supplementary Tables S1 and S2). Using logistic regression, we assessed the associations between parental IBD diagnoses and autism in children. We ran crude models (Model 1), as well as models adjusted for covariates that have been previously identified as associated with autism in the Swedish registers, including parental age at delivery^15^, migrant status^16^, education level, family income quintile at birth^17^, parents’ history of psychiatric diagnosis^18^ prior to the birth of the child, and child’s sex, birth year, and birth order (Model 2). In order to avoid potential bias from assortative mating in Model 2, we additionally mutually adjusted for maternal and paternal IBD diagnoses (Model 3)^19^. Maternal IBD diagnosis was associated with autism in children in crude and adjusted models (any IBD diagnosis: odds ratio (OR)_MODEL3_ = 1.32; 95% confidence intervals (CIs): 1.25 to 1.40; Table 2). Similar results were observed in analyses of maternal UC and Crohn’s diagnoses and autism in children (Table 2). The paternal IBD associations with autism were weaker (OR_MODEL3_ = 1.09; 95% CIs 1.02 to 1.17) than the maternal associations (Table 2). Results of the analysis were not sensitive to the choice of parental psychiatric history variable (broad psychiatric history versus parental diagnoses of autism specifically) or exclusion procedures that aimed to control for neurodeveloplmental outcomes that we assumed to have a genetic cause, though point estimates were lower in analyses restricted to parental IBD diagnoses prior to the index person’s birth (any maternal IBD diagnosis: OR_MODEL3_ = 1.20; 95% CIs: 1.09 to 1.32; Supplementary Table S3). Point estimates for associations of parental IBD diagnoses to autism without intellectual disabilities (IDs) were higher than those for autism with ID, although CIs overlapped (Supplementary Table S4).

**Table 2:** Associations between maternal or paternal diagnosis for any IBD, UC, Crohn’s, or other IBD, and diagnosis of autism in children

Exposure	*n* ASD/*N* total (% ASD)^a^	Model1^b^ OR (95% CIs)	*P*	Model2^c^ OR (95% CIs)	*P*	Model3^d^ OR (95% CIs)	*P*
No maternal IBD	43,568/2,272,606 (1.92%)	Ref		Ref		Ref	
Any maternal IBD	1,361/51,621 (2.64%)	1.39 (1.31,1.47)	<0.001^e^	1.32 (1.24,1.40)	<0.001^e^	1.32 (1.25,1.40)	<0.001^e^
Maternal Crohn’s	422/17,832 (2.37%)	1.23 (1.09,1.40)	0.001^e^	1.19 (1.05,1.35)	0.006	1.20 (1.06,1.36)	0.004
Maternal UC	292/12,390 (2.36%)	1.24 (1.12,1.38)	<0.001^e^	1.22 (1.10,1.35)	<0.001^e^	1.22 (1.10,1.36)	0.001
Maternal Other IBD^f^	722/24,865 (2.90%)	1.53 (1.42,1.66)	<0.001^e^	1.42 (1.32,1.54)	<0.001^e^	1.43 (1.32,1.55)	<0.001^e^
Maternal Crohn’s or UC^g^	639/26,756 (2.39%)	1.25 (1.15,1.35)	<0.001^e^	1.21 (1.11,1.32)	<0.001^e^	1.22 (1.12,1.32)	<0.001^e^
No paternal IBD	43,989/2,281,119 (1.93%)	Ref		Ref		Ref	
Any paternal IBD	940/43,108 (2.18%)	1.14 (1.06,1.22)	<0.001^e^	1.11 (1.03,1.18)	0.004	1.09 (1.02,1.17)	0.012
Paternal Crohn’s	346/18,290 (1.89%)	1.18 (1.04,1.35)	0.013	1.16 (1.02,1.33)	0.023	1.16 (1.01,1.32)	0.031
Paternal UC	254/11,274 (2.25%)	0.99 (0.88,1.10)	0.806	0.98 (0.87,1.09)	0.662	0.97 (0.86,1.08)	0.575
Paternal other IBD^f^	407/16,958 (2.40%)	1.25 (1.12,1.38)	<0.001^e^	1.19 (1.07,1.32)	0.001^e^	1.17 (1.05,1.30)	0.003
Paternal Crohn’s or UC^g^	533/26,150 (2.04%)	1.06 (0.97,1.16)	0.187	1.05 (0.96,1.15)	0.312	1.04 (0.95,1.14)	0.408

^a^The total numbers for those exposed to maternal or paternal Crohn’s, UC, or other IBD do not sum to the total exposed to any IBD because some mothers or fathers received both a Crohn’s and a UC diagnosis. Supplementary Fig. S2 shows details on the prevalence and overlap in diagnoses in the study sample.

^b^Crude models.

^c^Models adjusted for child’s sex, year of birth, birth order, maternal/paternal age, migrant status, education level, family income, and parental psychiatric history.

^d^Mutually adjusted models for maternal/paternal IBD diagnoses, child’s sex, year of birth, birth order, maternal/paternal age, migrant status, education level, family income, and parental psychiatric history.

^e^*P* value is less than Bonferroni-corrected value of 0.0012, accounting for 42 models in Study 1. Supplementary Table 19 shows exact *P* values.

^f^Excluding Crohn’s and UC and including ICD-9 558 ‘other and unspecified noninfectious gastroenteritis and colitis’ and ICD-10 K52.3 ‘indeterminate colitis’ and K52.9 ‘noninfective gastroenteritis and colitis.’ Extended Data Fig. 9 shows diagnostic code details.

ORs, 95% CIs, and *P* values were calculated using generalized estimating logistic models, with robust standard errors accounting for clustering of multiple children born to the same parents. ASD: autism spectrum disorder.

### Study 2: Genetic correlation between IBD and autism

Linkage disequilibrium score regression (LDSC) allows the estimation of the genetic correlation between complex traits such as IBD and autism by utilizing GWAS summary data^20,21^ (Table 1).

Using the latest GWAS summary statistics on IBD (*N*_cases_ = 25,042; *N*_controls_ = 34,915) (ref. ^22^), Crohn’s (*N*_cases_ = 12,194; *N*_controls_ = 28,072) (ref. ^22^), UC (*N*_cases_ = 12,366; *N*_controls_ = 33,609) (ref. ^22^), and autism (*N*_cases_ = 18,381; *N*_controls_ = 27,969) (ref. ^23^), we performed LDSC. We found no evidence of a genetic correlation between genetic liability to autism and IBD, UC, or Crohn’s (Table 3). Heritability scores (*z* scores: 8.34–11.75), chi-squares (1.20–1.53), and intercepts (1.01–1.12) satisfied the conditions to provide reliable LDSC estimates (Supplementary Table S5).

**Table 3:** Genetic correlation (*r*_g_) between liability to autism and IBD, UC, and Crohn’s

Trait 1	Trait 2	*r*_g_ (95% CIs)	*P*
Autism	IBD	−0.0615 (−0.15, 0.02)	0.158
Autism	UC	−0.0656 (−0.17, 0.04)	0.2064
Autism	Crohn’s	−0.0403 (−0.13, 0.05)	0.3551

Genetic correlation coefficients (*r*_g_), 95% CIs and *P* values calculated by LDSC. There were no multiple-test corrections made.

### Study 3: Polygenic risk for IBD and broad autistic traits

Polygenic Risk Score (PRS) approaches enable the estimation of an individual’s underlying genetic liability to a complex trait. PRSs require individual-level genotype data, and are calculated as the sum of the individual’s risk alleles, weighted by the effect sizes of each variant identified in the GWAS of the trait^24^. In the context of the present study, individual-level data from the Avon Longitudinal Study of Parents and Children (ALSPAC) were used^25–27^. PRS approaches are particularly important for the triangulation of evidence from traditional observational approaches, since they allow the refinement of the exposure used in the context of the observational study (that is, they can potentially overcome misclassification bias of an observational study (Table 1)^28^.

**Extended Data Fig. 4:**
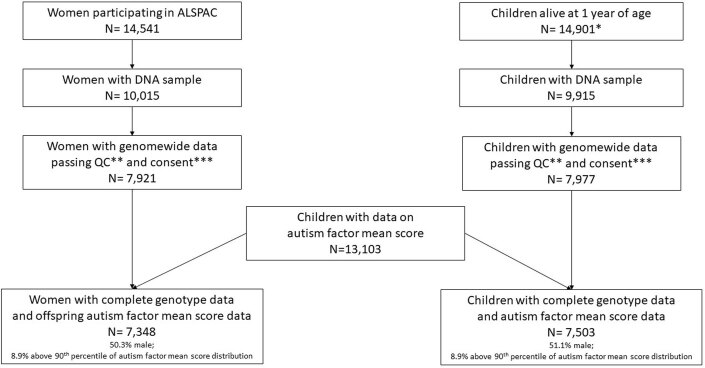
Study sample derivation and characteristics for the PRS analyses in ALSPAC. ^a^Although initial ALSPAC recruitment resulted in 13,988 children who were alive at 1 year of age, when the children were approximately 7 years old, the initial sample was bolstered with eligible children who did not join the study initially. 913 children were additionally enrolled during three phases of recruitment. This resulted in 14,901 children alive at 1 year of age. Detailed information can be found at references^24–26,64–66^ ^b^Details on QC process can be found in Methods. ^c^Consent for biological samples has been collected in accordance with the Human Tissue Act (2004).

In 7,348 mothers and 7,503 children of the ALSPAC cohort, we calculated PRSs for IBD, Crohn’s, and UC, using the latest available GWAS summary data^22^, and assessed associations with an available measure of broad autistic traits, autism mean factor score^29^ (Methods, Extended Data Fig. 4).

#### Maternal PRS for IBD and broad autistic traits in children

**Extended Data Fig. 5:**
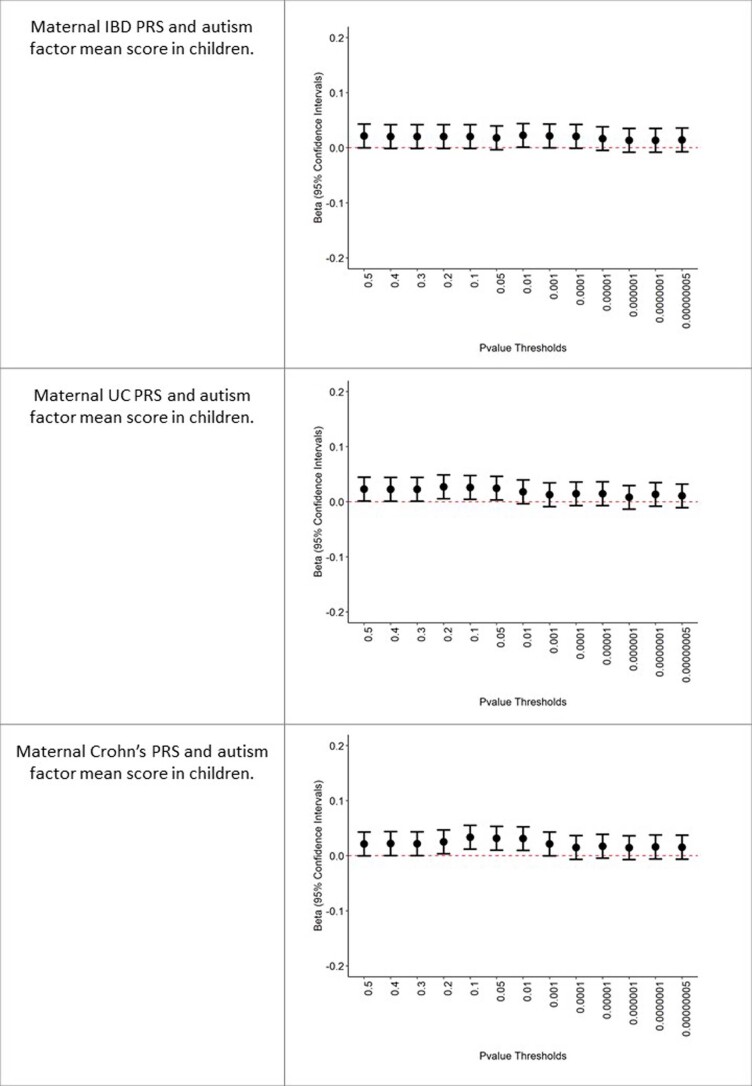
Associations between maternal polygenic risk score for IBD, UC, Crohn’s, and autism factor mean score in the children, at 13 *P*-value thresholds for PRS generation. *n* = 7,348 mother–child pairs. Data are presented as beta coefficients and 95% CIs. Calculated by linear regression of the mothers’ PRS for IBD, UC, or Crohn’s on the standardized autism factor mean score in the children, adjusted for child’s sex and the first ten principal components of the ALSPAC genotype data.

Maternal polygenic risk for UC and Crohn’s was associated with a higher autism factor mean score in the child (UC: *β*_PRS_ = 0.02; 95%CIs: 0.003 to 0.05; *P* = 0.03; Crohn’s: *β*_PRS_ = 0.03; 95%CIs: 0.01 to 0.05; *P* = 0.004). Similar results were found across other *P*-value thresholds (0.50–0.05). The effect size of the association between maternal polygenic risk for IBD and autism factor mean score, was comparable with that of UC and Crohn’s, although CIs crossed the null (*β*_PRS_ = 0.02; 95%CIs: −0.004 to 0.040; *P* = 0.1; *R*^2^ = 0.06; Table 4, Extended Data Fig. 5, Supplementary Table S6).

**Table 4:** Associations between maternal and child PRS for IBD, UC, Crohn’s at *P*-value threshold 0.05, and standardized autism factor mean score in the children of the ALSPAC birth cohort

Exposure	*β* (95% CIs)	*P*
Maternal IBD PRS	0.02 (−0.004, 0.04)	0.1
Maternal UC PRS	0.02 (0.003, 0.05)	0.03
Maternal Crohn’s PRS	0.03 (0.01, 0.05)	0.004
Child’s IBD PRS	0.003 (−0.02, 0.02)	0.79
Child’s UC PRS	0.001 (−0.02, 0.02)	0.89
Child’s Crohn’s PRS	0.007 (−0.01, 0.03)	0.49

*N* = 7,348 mother-child pairs for analyses involving mothers’ PRSs. *N* = 7,503 children for analyses involving children’s PRS. Beta coefficients, 95% CIs, and *P* values calculated by linear regression of the PRS for IBD, UC, or Crohn’s on the standardized autism factor mean score in the children, adjusted for child’s sex and the first ten principal components of the ALSPAC genotype data. Higher standardized autism factor mean scores (mean = 0, s.d. = 1) reflect more autism-related difficulties. There were no multiple-test corrections made.

#### Child’s PRS for IBD and broad autistic traits

**Extended Data Fig. 6:**
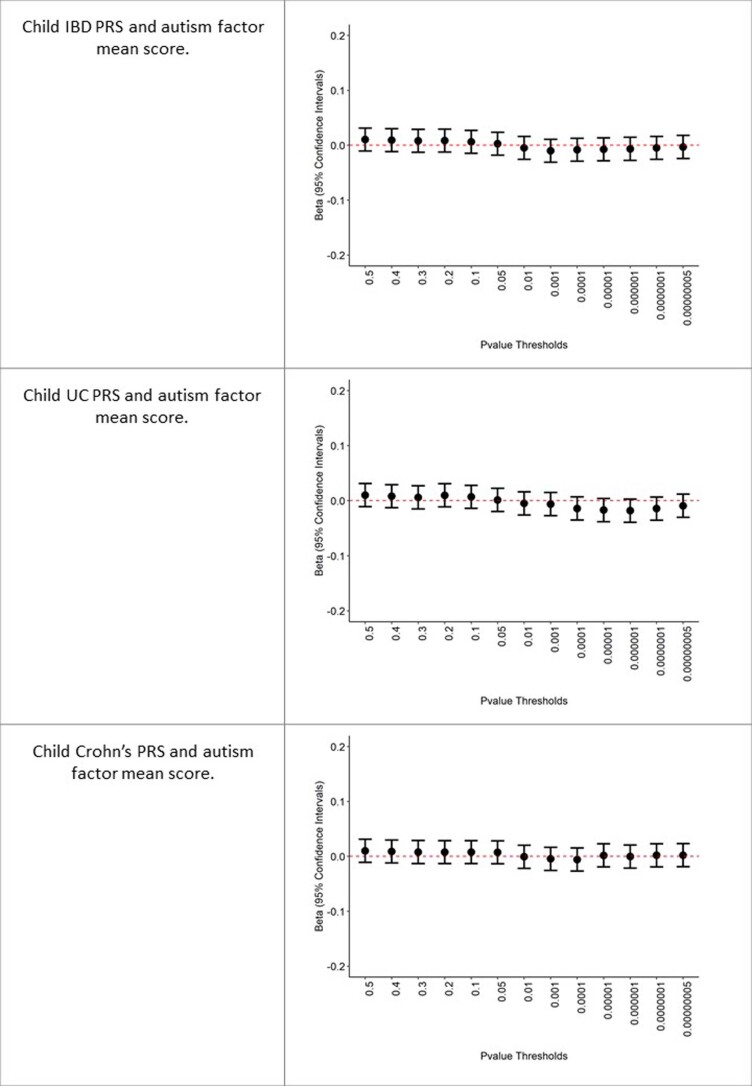
Associations between children’s polygenic risk score for IBD, UC, Crohn’s, and autism factor mean score, at 13 *P*-value thresholds for PRS generation. *n* = 7,503 children. Data are presented as beta coefficients and 95% CIs. Calculated by linear regression of the children’s PRS for IBD, UC, or Crohn’s on the standardized autism factor mean score in the children, adjusted for child’s sex and the first ten principal components of the ALSPAC genotype data.

There was no evidence of associations between a child’s PRS for IBD, UC, Crohn’s, and autism mean factor score in children (IBD: *β*_PRS_ = 0.003; 95%CIs: −0.02 to 0.02; *P* = 0.79; *R*^2^ = 0.05; UC: *β*_PRS_ = 0.001; 95%CIs: −0.02 to 0.02; *P* = 0.89; *R*^2^ = 0.05; Crohn’s: *β*_PRS_ = 0.007; 95%CIs: −0.01 to 0.03; *P* = 0.49; *R*^2^ = 0.05; Table 4, Extended Data Fig. 6, Supplementary Table S7).

### Study 4: Causal effect of genetic liability to IBD on autism

MR is a causal inference approach that can overcome limitations of observational and PRS approaches (Table 1). MR is based on the principles of instrumental variables analyses, utilizing germline genetic variants as instruments for exposures to assess their causal effects on outcomes of interest^30–32^. Since genetic variants are randomly assorted at meiosis and fixed at conception, the method is effective in minimizing confounding and reverse causation bias that hampers observational studies^32,33^. In contrast to PRS approaches that estimate associations, under certain assumptions that the instruments should satisfy, MR can generate unbiased causal effect estimates. The core assumptions of MR are outlined in the Methods.

**Extended Data Fig. 7:**
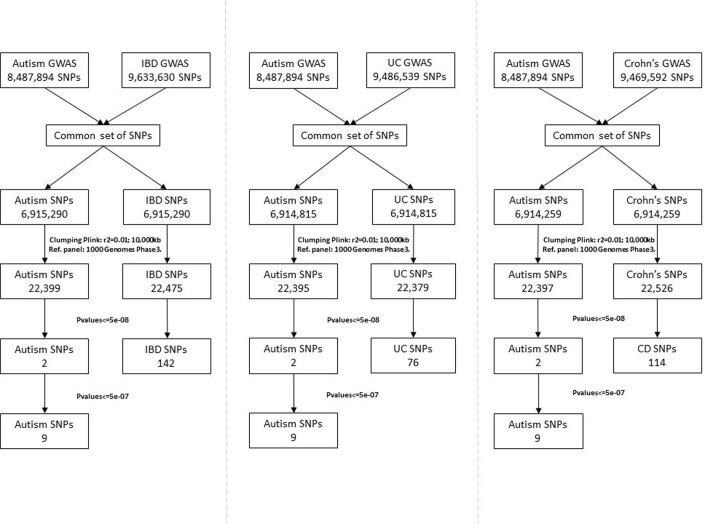
Genetic instrument extraction process for the MR analyses investigating the causal links between genetic liability to autism and IBD, UC, and Crohn’s. Between phenotype pairs, GWAS summary data were restricted to a common set of SNPs and then clumped in PLINK 1.90 using the 1000 Genomes phase 3 European ancestry reference panel, and an *r*^2^ = 0.01, within a 10,000 Kb window. Among the independent variants, instruments were defined using a genome-wide significance threshold of *P* ≤ 5×10^−8^. The only exception was autism, as only two independent and genome-wide significant variants were identified. We therefore relaxed the *P*-value threshold to 5 × 10^−7^ to improve statistical power, as used previously^72^).

**Extended Data Fig. 8:**
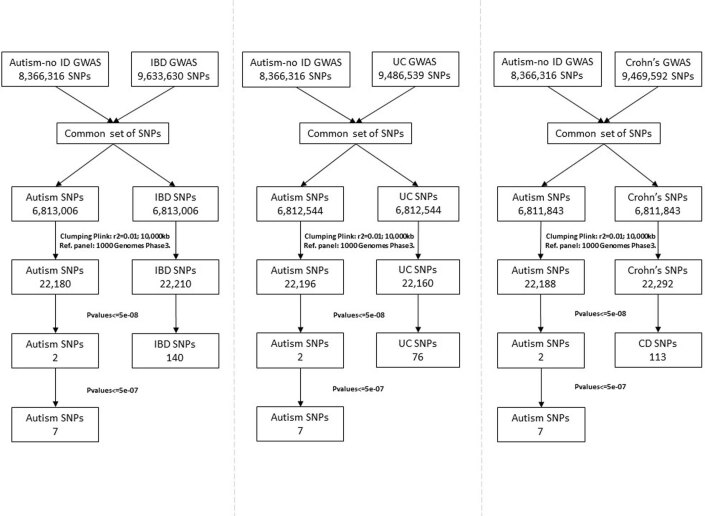
Genetic instrument extraction process for the MR analyses investigating the causal links between genetic liability to autism without ID (iPSYCH subsample) and IBD, UC, and Crohn’s. Between phenotype pairs, GWAS summary data were restricted to a common set of SNPs and then clumped in PLINK 1.90 using the 1000 Genomes phase 3 European ancestry reference panel, and an *r*^2^ = 0.01, within a 10,000 Kb window. Among the independent variants, instruments were defined using a genome-wide significance threshold of *P* ≤ 5 × 10^−8^. The only exception was autism, as only two independent and genome-wide significant variants were identified. We therefore relaxed the *P*-value threshold to 5 × 10^−7^ to improve statistical power, as used previously^72^.

Within a two-sample MR^33^ framework, we extracted common genetic variants robustly associated (*P* ≤ 5.0^−8^) with IBD, Crohn’s, and UC using the latest available GWAS summary data^22^, and assessed their causal effects on 18,381 autism cases and 27,969 controls of the PGC and the iPSYCH consortia^23^ (Online Methods, Extended Data Fig. 7, Supplementary Table S8). MR analyses were additionally performed using a subsample of the iPSYCH excluding all ID cases (*N*_cases_ = 11,203; *N*_controls_ = 22,555; Methods, Extended Data Fig. 8, Supplementary Table S9).

The mean *F* statistics of the IBD, UC, and Crohn’s instruments were 67, 68, and 70, respectively, suggesting adequate strength^34^. There was evidence of a causal effect of genetic liability to UC on risk of autism (_IVW_OR = 1.04; 95% CIs: 1.01 to 1.07; *P* = 0.006). Evidence for the effect of genetic liability to IBD and Crohn’s on autism risk was weaker, although the magnitude and direction of the effect estimates was comparable with the UC results (Table 5).

**Table 5:** Mendelian randomization IVW estimates for the effect of genetic liability to IBD, Crohn’s, and UC on autism and vice versa

Exposure	Outcome	OR (95% CIs)	*P*
Genetic liability to IBD	Autism	1.02 (1.0, 1.05)	0.1
Genetic liability to UC	Autism	1.04 (1.01, 1.07)	0.006
Genetic liability to Crohn’s	Autism	1.01 (1.0, 1.04)	0.2
Genetic liability to autism	IBD	0.90 (0.73, 1.11)	0.32
Genetic liability to autism	UC	0.95 (0.77, 1.18)	0.65
Genetic liability to autism	Crohn’s	0.85 (0.63, 1.15)	0.29

ORs, 95% CIs, and *P* values calculated by inverse variance-weighted (IVW) Mendelian randomization. There were no multiple-test corrections made.

The magnitude and direction of causal effect estimates were consistent across all sensitivity analyses, and there was no evidence to suggest the influence of horizontal pleiotropy (Supplementary Table S10). Results of analyses with instruments extracted from the autism GWAS excluding ID cases were comparable with our primary effect estimates (Supplementary Table S11).

#### Causal effects of genetic liability to autism on risk of IBD

We assessed the possibility of reverse causation by performing bidirectional two-sample MR. We extracted common genetic variants associated (*P* ≤ 5.0^−7^) with autism, as well as autism without ID^23^, and assessed their potential causal effects on IBD (*N*_cases_ = 25,042; *N*_controls_ = 34,915), UC (*N*_cases_ = 12,366; *N*_controls_ = 33,609), and Crohn’s (*N*_cases_ = 12,194; *N*_controls_ = 28,072) (ref. ^22^) (Online Methods, Extended Data Figs. 6 and 7, Supplementary Tables S8 and S9). The mean *F* statistic of the autism instruments was 28, suggesting adequate strength. There was no evidence of a causal effect of genetic liability to autism on risk of IBD, UC, or Crohn’s (Table 5). The estimates were consistent across sensitivity analyses, with overlapping confidence intervals, and were unlikely to be influenced by horizontal pleiotropy (Supplementary Table S12). Repeating our analyses with instruments extracted from the autism GWAS excluding all ID cases yielded similar results (Supplementary Table S13).

## Discussion

We used four complementary approaches to investigate the associations between parental diagnoses and genetic liability to IBD and autism in children. On conducting a nationwide register-based cohort study in Sweden we found evidence of associations between parental diagnoses of IBD and autism in children. Importantly, the maternal effect sizes were larger than the paternal sizes, without overlapping CIs. PRS analyses in the ALSPAC birth cohort suggested associations between maternal genetic liability to IBD and autism traits in children, while two-sample MR studies provided evidence of a potential causal effect of genetic liability to IBD on autism risk. There was no evidence to suggest a genetic correlation between autism and IBD, as indicated by LDSC analyses.

A number of studies have investigated the potential associations between parental autoimmune conditions and autism. Several parental autoimmune conditions have been previously identified as linked to autism in children, including rheumatoid arthritis^35^ and psoriasis^36^. In the case of IBD, evidence from previous studies is inconclusive. In contrast to studies to date, the use of four distinct study designs is a notable strength of our approach. Using study designs with different strengths and sources of bias (Table 1) allowed the triangulation of our findings, rather than relying on arbitrary *P*-value thresholds^28,37^. The Swedish nationwide register-based cohort study of over two million parent–child pairs is the largest to date on parental IBD and autism in children. In addition, the present study benefited from the longest to date follow-up period (1987–2016), as well as exposure and outcome ascertainment from both inpatient and outpatient specialist care.

The ALSPAC cohort containing genotype data for over 7,000 mothers and children, as well as broad autistic trait measures for over 13,000 children, is one of the richest resources for the investigation of the potential polygenic associations between maternal polygenic risk for IBD and autism in children. Finally, in the MR analyses, we used the largest GWAS data available for all conditions and conducted several sensitivity analyses to test the robustness of our findings.

Considering study limitations in the Swedish registers, the possibility of measurement error in IBD diagnoses cannot be excluded. However, this is likely to be nondifferential in relation to our study outcome and would therefore bias our findings towards the null. Second, while PRSs were based on large GWAS samples, it was not possible for us to investigate the variance explained by the PRSs in our target sample. However, based on previous studies^38,39^, it could be expected that our PRSs potentially explain little variance in the phenotype (≈1.5–3.0%), a limitation that could be overcome with future larger GWAS. Third, the autism mean factor score used in the present analyses was derived from individual measures that were not primarily intended to assess autism. However, the score has been found predictive of a clinical autism diagnosis (measured independent of the variables contributing to the derivation of the mean factor score) and presents associations with autism PRS in ALSPAC, as suggested by previous studies^29,40^. Fourth, in two-sample MR analyses investigating the effects of genetic liability to autism on risk of IBD, we used a relaxed instrument inclusion *P*-value threshold, this could potentially result in including weak instruments and therefore bias the causal effect estimates. The *F* statistic of the autism instruments in our analyses suggested that weak instrument bias is unlikely. Fifth, although we performed a series of sensitivity analyses to assess the robustness of the causal effect estimates, the possibility of horizontal pleiotropy influencing the present findings cannot entirely be ruled out, especially considering emerging evidence on the genetic architecture of IBD, implicating immune and endocrine-related genes^41^. Sixth, using GWAS data, we could only investigate the possible contribution of common variants acting under an additive model and not any contribution from rare variation which is found to be implicated in autism^42,43^. Finally, an important consideration is that the present study has been conducted using samples and GWAS data of predominantly European ancestry individuals. Although a proportion of index children in the registry-based study had at least one parent of non-European descent (10%), the use of European ancestry summary and individual-level genetic data in LDSC, PRS, and MR analyses, was unavoidable considering the largest available GWAS on autism and IBD has been conducted in European ancestry samples. The increasing representation of ancestrally diverse populations in biobanks and health registers will allow future studies to build on the present findings.

Overall, our findings suggest larger maternal effect sizes than paternal in the registry-based study, in combination with the identified associations between maternal, but not child’s, PRS for IBD and child’s autism factor mean score, which could potentially indicate in-utero effects. This could be further supported considering we did not find evidence of a genetic correlation between autism and IBD. Specifically, based on liability-threshold models of inheritance^44–47^ (and assuming that liability to IBD is normally distributed in the population), it could be hypothesized that liability to IBD will be expressed after a threshold has been exceeded, depending on a synergy of genetic variation, environmental factors, and chance. Mothers close to the threshold, but not exceeding it, could be expected to express subphenotypic manifestations of IBD, such as immunological alterations, micronutrient deficiencies, or anemia. These subphenotypic manifestations could influence fetal development. In fact, several immune pathways have been implicated in both Crohn’s and UC (which are strongly genetically correlated: *r*_g_ = 0.5; *P* = 2.0 × 10^−^^13^ (ref. ^20^)), including T-helper 1, T-helper 2, and T-helper 17 cytokines^48^, which are increasingly identified as linked to perinatal complications^49–51^, as well as autism^52–54^. Similarly, micronutrient malabsorption and anemia during pregnancy have been found to be associated with autism in children^9,10^. The availability of genotype and biospecimen data in autism family cohorts such as the Simons Simplex Collection and the Simons Foundation Powering Autism Research (SPARK)^55,56^, is expected to allow the integration of genomic, immune, and gut microbiome profiling approaches to elucidate the potential etiology and biological pathways underlying the identified associations.

In conclusion, triangulating evidence from a nationwide register-based cohort study, genetic correlation, PRS analyses, and MR, we found evidence suggesting associations between parental, particularly maternal, diagnoses of IBD, and autism in children. Links between maternal genetic liability to IBD and autism in children may reflect the influence of the maternal genotype on the prenatal/intrauterine environment. Investigating the mechanisms behind these findings may provide valuable insights into the origins of autism.

## Methods

Throughout the text, the terms autism and autistic people/individuals are used, in line with recent evidence suggesting that these terms are preferred in the autistic community and are less stigmatizing^58,59^.

### Study 1: Swedish cohort study

We used individual-level data from ‘Psychiatry Sweden’ to investigate whether parental IBD diagnosis is associated with autism diagnosis in children. ‘Psychiatry Sweden’ is a comprehensive national register linkage, with approval from the Stockholm regional ethical review committee (DNR 2010/1185-31/5, 2016/987-32). In line with the standards of all register-based research in Sweden and in keeping with the specific ethical approval for ‘Psychiatry Sweden’, informed patient consent was not required for the analysis of the anonymized data.

All children born in Sweden from 1 January 1987 to 31 December 2010 (*N* = 2,837,045) were eligible index persons, with follow up to 31 December 2016. Exclusion criteria were: children born outside Sweden (*n* = 292,023), children not registered in the Medical Birth Register (*n* = 74,240), children resident in Sweden for under 5 years (*n* = 23,495), children of multiple pregnancy (*n* = 67,309), children who were adopted (*n* = 2,425), children who received a diagnosis of autism or ID who also had a documented genetic/metabolic condition known to cause neurodevelopmental disorders (for example, trisomies) (*n* = 7,873), or incomplete parental records (*n* = 45,453) (ref. ^60^). The study population included 2,324,227 children born to 1,282,494 mothers and 1,285,719 fathers (Extended Data Fig. 1).

**Extended Data Fig. 9:**
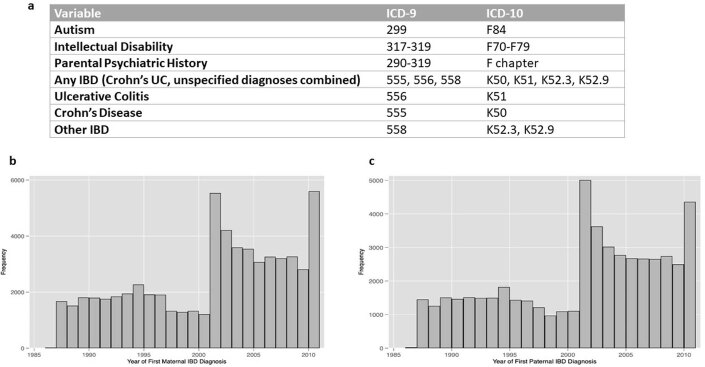
Ascertainment of outcomes and exposures from the National Patient Register (NPR). **a,** Diagnostic codes for ascertainment of outcomes and exposures. **b,** Frequency of IBD diagnoses, by year, in NPR for study cohort mothers from 1987 to 2010. **c,** Frequency of IBD diagnoses, by year, in NPR for study cohort fathers from 1987 to 2010. ICD-9: *International Classification of Diseases* – *ninth edition*; ICD-10: *International Classification of Diseases – tenth edition*.

The National Patient Register (NPR) includes inpatient care records beginning in 1973, outpatient physician visits in specialist care from 1997, outpatient psychiatric diagnoses from 2006, and children and adolescent psychiatric care from 2011. Autism was identified in the National Patient Register (NPR) using ICD-9 and ICD-10 codes (Extended Data Fig. 9). Lifetime history of parental IBD, Crohn’s disease (Crohn’s) and ulcerative colitis (UC) were identified using ICD-9 and ICD-10 codes in the NPR (Extended Data Fig. 9). We used parental lifetime IBD diagnosis as the primary exposure. This approach was considered appropriate since data from outpatient specialist care were not originally included in the NPR and these were added starting in the late 1990s. Extended Data Fig. 9 illustrates the frequency of IBD diagnoses (for mothers and fathers of the study cohort) in NPR from 1987 to 2010.

Using STATA/MP17, we estimated the odds ratios and 95% CIs of the association of mother’s and father’s diagnosis of IBD (any IBD, Crohn’s, or UC) with autism in children using generalized estimating logistic models with robust standard errors accounting for clustering of multiple children born to the same parents.

Model 1 was unadjusted. Model 2 was adjusted for parental age at delivery^15^, migrant status^16^, education level, family income quintile at birth^17^, parents’ history of psychiatric diagnosis prior to the birth of the child, and child’s sex, birth year, and birth order (Supplementary Table S14 for collinearity diagnostics of covariates included in the models). Model 3 was additionally mutually adjusted for maternal and paternal IBD diagnoses to avoid bias from assortative mating^19^. Additionally, we investigated associations between any parental IBD diagnoses and autism in children with and without ID separately, since these groups may have distinct genetic and environmental risk factors^18,61–63^ and outcomes^64,65^. Due to the number of analyses run in the study, we applied a Bonferroni correction to account for multiple testing (0.05/42 = 0.0012). We compared the results of three sensitivity analyses with the results of the main analysis. First, we restricted parental IBD diagnoses to those recorded prior to the birth of the index person. Second, we adjusted Models 2 and 3 for parental lifetime autism diagnoses specifically, instead of the broad definition of parental psychiatric history used in the main analysis. Finally, we repeated the analyses without exclusion of the 7,873 children who had a documented genetic/metabolic condition assumed to be causing their neurodevelopmental disorder.

### Study 2: LDSC

We used LDSC to estimate the genetic correlation between genetic liability to autism and IBD, Crohn’s, and UC.

LDSC allows the estimation of the genetic correlation between polygenic traits using GWAS summary statistics by capitalizing on patterns of linkage disequilibrium among common genetic variants^20^. We used the latest available GWAS summary data on autism (*N*_cases_ = 18,381; *N*_controls_ = 27,969) (ref. ^23^), IBD (*N*_cases_ = 25,042; *N*_controls_ = 34,915) (ref. ^22^). Crohn’s (*N*_cases_ = 12,194; *N*_controls_ = 28,072) (ref. ^22^), and UC (*N*_cases_ = 12,366; *N*_controls_ = 33,609) (ref. ^22^). Detailed information on study samples and case definition can be found in the original publications.

We followed the suggested protocol for LDSC analyses (https://github.com/bulik/ldsc/wiki). Using the LDSC (LD score) v.1.0.1 software in Python v.2.7.18, we estimated genetic correlations using pre-computed LD scores from the 1000 Genomes project European data^66^ (from: https://data.broadinstitute.org/alkesgroup/LDSCORE/eurwld_chr.tar.bz) with an unconstrained intercept term to account for any sample overlap and population stratification.

Ethics committee approval was not required for this analysis of publicly available GWAS summary statistics.

### Study 3: Polygenic Risk Score analyses in the ALSPAC cohort

#### Discovery sample

Common genetic variants, corresponding alleles, effect sizes, and *P* values were extracted to calculate PRSs from the GWAS summary data of IBD^22^, UC^22^, and Crohn’s^22^ described above.

#### Target sample

ALSPAC is a UK prospective birth cohort study based in Bristol and surrounding areas, which recruited pregnant women with expected delivery dates from 1 April 1991 to 31 December 1992; 14,541 women were initially enrolled, with 14,062 children born, and 13,988 children alive at 1 year of age. Detailed information on the cohort is available elsewhere^25–27^. A fully searchable study data dictionary is available at: http://www.bristol.ac.uk/alspac/researchers/our-data/. Ethical approval for the study was obtained from the ALSPAC Ethics and Law Committee and the Local Research Ethics Committees.

##### Genetic data

10,015 ALSPAC mothers were genotyped on the Illumina Human660W quad genome-wide single nucleotide polymorphism (SNP) genotyping platform at the Center National Genotypage, and genotypes were identified using Illumina GenomeStudio. A total of 9,912 ALSPAC children were genotyped on the Illumina HumanHap550 quad chip genotyping platforms by 23andme subcontracting the Wellcome Trust Sanger Institute, Cambridge, UK, and the Laboratory Corporation of America, Burlington, NC, United States.

PLINK v1.07 was used for quality control filtering^67^. Specifically, individuals were excluded on the basis of the following filters: (1) gender mismatches; (2) undetermined X chromosome heterozygosity; (3) over 3% missingness (children); over 5% missingness (mothers); (4) evidence of crypted relatedness (>10% of shared alleles identical by descent in children and >12.5% of shared alleles identical by descent in mothers); (5) non-European ancestry, assessed by multidimensional scaling analysis compared with HapMap 2 individuals. SNPs were excluded on the basis of the following filters: (1) minor allele frequency < 1%; (2) call rate < 95%, (3) Hardy–Weinberg equilibrium (HWE) *P* < 5.0 × 10^−7^. Maternal and offspring genotype data were combined and imputed using Impute v.2.2.2 against 1000 Genomes reference panel (v.1, phase 3, December 2013 release).

After quality control and excluding participants who had withdrawn consent, genetic data were available for 7,921 mothers and 7,977 children of European ancestry. Consent for biological samples was collected in accordance with the Human Tissue Act (2004).

##### Broad autistic traits: autism factor mean score

We used a measure of broad autistic traits previously estimated in ALSPAC as the mean score of seven factors derived from a factor analysis of 93 measures related to autism in ALSPAC^29^. The measure was available in 13,103 children, and strongly predictive of the autism diagnosis measured independently via school records, record linkage, and parental reports^29^. Other autism trait measures or diagnoses were not used, as there were fewer genotyped mothers and children with these measures.

##### Calculation of PRSs in ALSPAC and statistical analysis

PRSs were calculated using PLINK v.1.9, applying the method described by the Psychiatric Genomics Consortium (PGC)^68^. SNPs with mismatching alleles between the discovery and target dataset were removed. The Major Histocompatibility Complex (MHC) region was removed (25–34 Mb), except for one SNP representing the strongest signal within the region. Using ALSPAC data as the reference panel, SNPs were clumped with an *r*^2^ of 0.25 and a physical distance threshold of 500 kB. The optimal *P*-value threshold for PRS is dependent on discovery and target sample sizes, as well as SNP inclusion parameters (for example, *r*^2^) (refs. ^24,69^). For this reason, we calculated PRS for each participant across 13 *P*-value thresholds (*P* < 5.0 × 10^−8^ to *P* < 0.5), standardized by subtracting the mean and dividing by the standard deviation. We defined PRS corresponding to *P*-value threshold 0.05 as our primary exposure, based on a previous ALSPAC study^70^. This threshold has been found to have sufficient predictive ability for IBD and its subtypes^39^. We could not directly assess the predictive power and optimal *P*-value threshold of the PRSs in our target sample, as there were few UC (*n* = 12) and Crohn’s cases (*n* = 16).

After constructing PRSs for IBD, UC, and Crohn’s in mothers and children, we performed linear regressions using STATA/MP 15 to examine associations with the standardized autism factor mean score in childhood. Analyses were adjusted for child’s sex and the first ten principal components of the ALSPAC genotype data to avoid population stratification bias^24^.

### Study 4: Two-sample MR

We performed two-sample MR to assess bidirectional causal links between genetic liability to autism and IBD and its subtypes, and vice versa.

MR can be implemented as an instrumental variable approach, utilizing common genetic variants as instruments for exposures of interest, allowing assessment of causal effects and their direction on outcomes. MR relies on the following assumptions: (1) there must be a robust association between the common genetic variants and the exposure (that is, no horizontal pleiotropy, the phenomenon in which the genetic variant influences multiple phenotypes through biologically distinct pathways); (2) the variants should operate on the outcome entirely via the exposure; and (3) the variants should not be associated with any confounders of associations between exposure and outcome^71^. In this study, we applied two-sample MR, in which the effect sizes and standard errors of the instruments for the exposure and the outcome were extracted from separate GWASs conducted in independent samples from the same underlying population^33^.

#### Genetic instruments

Genetic instruments were extracted from the overlapping set of SNPs between the autism^23^, IBD^22^, UC^22^, and Crohn’s^22^ GWASs. This ensured that all selected genetic instruments would be present in the outcome GWAS.

GWAS summary data were restricted to a common set of SNPs and then clumped in PLINK 1.90 using the 1000 Genomes^66^ phase 3 European ancestry reference panel, and an *r*^2^ = 0.01, within a 10,000 Kb window. Among the independent variants, instruments were defined using a genome-wide significance threshold of *P* ≤ 5 ×10^−8^. The only exception was autism, as only two independent and genome-wide significant variants were identified. We therefore relaxed the *P*-value threshold to 5 × 10^−7^ to improve statistical power, as used previously^72^. Extended Data Fig. 7 illustrates the process of instrument definition, and Supplementary Table S8 contains information on the genetic instruments used.

#### Harmonization

We harmonized the alleles of the outcome on the exposure, to ensure SNP-exposure and SNP-outcome effects correspond to the same allele. Variants identified as palindromic were removed, as the effect allele frequencies in the IBD, UC, and Crohn’s GWASs were not provided. Supplementary tables S15 and S16 contain details of the harmonized datasets.

#### Inverse Variance Weighted MR

The primary MR analysis was the inverse variance weighted (IVW) method which provides an overall causal effect estimate of the exposure on the outcome, estimated as a meta-analysis of the ratios of the SNP-outcome effect to the SNP-exposure effect weighted by each SNP’s relative precision^73^.

#### Sensitivity analyses

We assessed the strength of the instruments by estimating the mean *F* statistic. As a rule of thumb, the IVW is unlikely to suffer from weak instrument bias if mean *F* > 10 (ref. ^34^).

We assessed the consistency of the IVW causal effect estimates using sensitivity analyses, including MR Egger regression^73^, weighted median^74^, and weighted mode^75^ (Supplementary Table 20).

The autism GWAS used in our primary analyses included a proportion of autism cases with ID^23^. We tested the consistency of the causal effect estimates using GWAS summary data on a subsample of the iPSYCH cohort^76^ excluding all intellectual disability cases (*N*_cases_ = 11,203; *N*_controls_ = 22,555). Extended Data Fig. 8 visualizes the process of instrument definition, and Supplementary Tables S9, S17 and S18 contain details on the instruments used and the harmonized datasets.

Two-sample MR analyses were performed using the TwoSampleMR R package^77^ in R v.3.5.1. Ethics committee approval was not required for this analysis of GWAS summary statistics.

### Reporting summary

Further information on research design is available in the Nature Research Reporting summary linked to this article.

## Online content

Any methods, additional references, Nature Research reporting summaries, source data, extended data, supplementary information, acknowledgements, peer review information; details of author contributions and competing interests; and statements of data and code availability are available at 10.1038/s41591-022-01845-9.

## Supplementary information

Supplementary Information Supplementary Note.

Reporting Summary

Supplementary Tables Supplementary Tables 1–20.

## Data Availability

Swedish registry data: Individual-level data from ‘Psychiatry Sweden’ were used and under ethics approval from the Stockholm regional ethical review committee (DNR 2010/1185-31/5, 2016/987-32). Due to the sensitive nature of the data, data are not publicly available. Data must remain in the country, according to national laws and registry regulations. Access is restricted to projects approved by the Swedish ethical review authority (https://etikprovningsmyndigheten.se/) and in agreement with the register holders. See https://www.registerforskning.se/en/ for guidance on how to conduct Swedish register-based research. Since there is no central access point for public authority data in Sweden, this process may require coordination with multiple register holders (for example, Statistics Sweden, The National Board of Health and Welfare) and requires, in our experience, at least 1 year from the time of ethical approval, depending on workload for each register holder. GWAS summary data: GWAS summary data for IBD, UC, Crohn’s, and autism used in the LDSC, PRS and MR analyses, are publicly available (IBD: http://ftp.ebi.ac.uk/pub/databases/gwas/summary_statistics/GCST004001-GCST005000/GCST004131/; UC: http://ftp.ebi.ac.uk/pub/databases/gwas/summary_statistics/GCST004001-GCST005000/GCST004133/; Crohn’s: http://ftp.ebi.ac.uk/pub/databases/gwas/summary_statistics/GCST004001-GCST005000/GCST004132/; autism: https://www.med.unc.edu/pgc/download-results/). Restrictions apply to the availability of the GWAS summary data for autism without IDs, in order to ensure that there is no conflict with ongoing projects, collaborations and iPSYCH’s data-sharing policies. Data can be accessed after correspondence with the iPSYCH: https://ipsych.dk/. Researchers will be asked to prepare a short application, briefly describing the proposed study, and responses will typically be within 2 weeks. ALSPAC data: Ethical approval for the study was obtained from the ALSPAC Ethics and Law Committee and the Local Research Ethics Committees. Individual-level data from the ALSPAC birth cohort are not publicly available for reasons of clinical confidentiality. Data can be accessed after application to the ALSPAC Executive Team who will respond within 10 working days. Application instructions and data use agreements are available at http://www.bristol.ac.uk/alspac/researchers/access/. The minimum dataset for MR analyses is available in Supplementary Tables 8, 9, 15, 16, 17, and 18.

## Code availability

Analyses were conducted using established protocols for each analytic approach used in the present study. Specifically, in the case of LDSC, the protocol described at: https://github.com/bulik/ldsc/wiki/Heritability-and-Genetic-Correlation was used. In the case of PRS calculation, the approach described at: https://www.nature.com/articles/nature13595 was applied. Finally, for two-sample MR, the approach described at: https://mrcieu.github.io/TwoSampleMR/articles/introduction.html was applied.

## References

[1] Mubashir S, Farrugia M, Coretti L, Pessia M, D’adamo MC (2020). Autism spectrum disorder. Malta Med. J..

[2] Holingue C, Newill C, Lee LC, Pasricha PJ, Daniele Fallin M (2018). Gastrointestinal symptoms in autism spectrum disorder: a review of the literature on ascertainment and prevalence. Autism Res..

[3] Lee M (2018). Association of autism spectrum disorders and inflammatory bowel disease. J. Autism Dev. Disord..

[4] Graham DB, Xavier RJ (2020). Pathway paradigms revealed from the genetics of inflammatory bowel disease. Nature.

[5] Yoon SM (2016). Micronutrient deficiencies in inflammatory bowel disease: trivial or crucial?. Intest. Res..

[6] Weisshof R, Chermesh I (2015). Micronutrient deficiencies in inflammatory bowel disease. Curr. Opin. Clin. Nutr. Metab. Care.

[7] Osokine I, Erlebacher A (2017). Inflammation and autism: from maternal gut to fetal brain. Trends Mol. Med..

[8] Devilbiss EA (2017). Antenatal nutritional supplementation and autism spectrum disorders in the Stockholm youth cohort: population based cohort study. Brit. Med. J..

[9] Tan M (2020). Maternal folic acid and micronutrient supplementation is associated with vitamin levels and symptoms in children with autism spectrum disorders. Reprod. Toxicol..

[10] Wiegersma AM, Dalman C, Lee BK, Karlsson H, Gardner RM (2019). Association of prenatal maternal anemia with neurodevelopmental disorders. JAMA Psychiatry.

[11] Keil A (2010). Parental autoimmune diseases associated with autism spectrum disorders in offspring. Epidemiology.

[12] Atladóttir HÓ (2009). Association of family history of autoimmune diseases and autism spectrum disorders. Pediatrics.

[13] Andersen ABT, Ehrenstein V, Erichsen R, Frøslev T, Sørensen HT (2014). Autism spectrum disorders in children of parents with inflammatory bowel disease–a nationwide cohort study in Denmark. Clin. and Exp. Gastroenterol..

[14] Mouridsen SE, Rich B, Isager T, Nedergaard NJ (2007). Autoimmune diseases in parents of children with infantile autism: A case-control study. Dev. Med. Child Neurol..

[15] Idring S (2014). Parental age and the risk of autism spectrum disorders: findings from a Swedish population-based cohort. Int. J. Epidemiol..

[16] Magnusson C (2012). Migration and autism spectrum disorder: population-based study. Br. J. Psychiatry.

[17] Rai D (2012). Parental socioeconomic status and risk of offspring autism spectrum disorders in a Swedish population-based study. J. Am. Acad. Child Adolesc. Psychiatry.

[18] Xie S (2020). The familial risk of autism spectrum disorder with and without intellectual disability. Autism Res..

[19] Madley-Dowd P, Rai D, Zammit S, Heron J (2020). Simulations and directed acyclic graphs explained why assortative mating biases the prenatal negative control design. J. Clin. Epidemiol..

[20] Bulik-Sullivan BK (2015). LD score regression distinguishes confounding from polygenicity in genome-wide association studies. Nat. Genet..

[21] Bulik-Sullivan B (2015). An atlas of genetic correlations across human diseases and traits. Nat. Genet..

[22] De Lange KM (2017). Genome-wide association study implicates immune activation of multiple integrin genes in inflammatory bowel disease. Nat. Genet..

[23] Grove J (2019). Identification of common genetic risk variants for autism spectrum disorder HHS Public Access Author manuscript. Nat. Genet..

[24] Choi SW, Mak TSH, O’Reilly PF (2020). Tutorial: a guide to performing polygenic risk score analyses. Nat. Protoc..

[25] Fraser A (2013). Cohort profile: the Avon Longitudinal Study of Parents and Children: ALSPAC mothers cohort. Int. J. Epidemiol..

[26] Boyd A (2013). Cohort profile: The ‘Children of the 90s’-The index offspring of the Avon Longitudinal Study of Parents and Children. Int. J. Epidemiol..

[27] Northstone K (2019). The Avon Longitudinal Study of Parents and Children (ALSPAC): an update on the enrolled sample of index children in 2019 [version 1; peer review: 2 approved]. Wellcome Open Res..

[28] Lawlor DA, Tilling K, Davey Smith G (2016). Triangulation in aetiological epidemiology. Int. J. Epidemiol..

[29] Steer CD, Golding J, Bolton PF (2010). Traits contributing to the autistic spectrum. PLoS One.

[30] Sanderson E (2022). Mendelian randomization. Nat. Rev. Methods Prim..

[31] Davey Smith G, Hemani G (2014). Mendelian randomization: genetic anchors for causal inference in epidemiological studies. Hum. Mol. Genet..

[32] Davey Smith G, Ebrahim S (2003). ‘Mendelian randomization’: can genetic epidemiology contribute to understanding environmental determinants of disease?. Int. J. Epidemiol..

[33] Davey Smith G, Hemani G (2014). Mendelian randomization: Genetic anchors for causal inference in epidemiological studies. Hum. Mol. Genet..

[34] Pierce BL, Burgess S (2013). Efficient design for Mendelian randomization studies: subsample and 2-sample instrumental variable estimators. Am. J. Epidemiol..

[35] Rom AL (2018). Parental rheumatoid arthritis and autism spectrum disorders in offspring: a Danish nationwide cohort study. J. Am. Acad. Child Adolesc. Psychiatry.

[36] Croen LA, Grether JK, Yoshida CK, Odouli R, De Water JVan (2005). Maternal autoimmune diseases, asthma and allergies, and childhood autism spectrum disorders: a case-control study. Arch. Pediatr. Adolesc. Med..

[37] Sterne JAC, Davey Smith G, Cox DR (2001). Sifting the evidence—what’s wrong with significance tests?. Brit. Med. J..

[38] Glanville KP, Coleman JRI, O'Reilly PF, Galloway J, Lewis CM (2021). Investigating pleiotropy between depression and autoimmune diseases using the UK Biobank. BP:GOS.

[39] Chen, G. B. et al. Performance of risk prediction for inflammatory bowel disease based on genotyping platform and genomic risk score method. *BMC Med. Genet*. **18**, 94 (2017). 10.1186/s12881-017-0451-2 PMC5576242 28851283

[40] Rai D (2018). Association of autistic traits with depression from childhood to age 18 years. JAMA Psychiatry.

[41] Lasconi C (2021). Variant-to-gene-mapping analyses reveal a role for the hypothalamus in genetic susceptibility to inflammatory bowel disease. CMGH.

[42] Buxbaum JD (2009). Multiple rare variants in the etiology of autism spectrum disorders. Dialogues Clin. Neurosci..

[43] Balzola F, Bernstein C, Ho GT, Russell RK (2012). Deep resequencing of GWAS loci identifies independent rare variants associated with inflammatory bowel disease: commentary. Inflamm. Bowel Dis. Monit..

[44] Dempster ER, Lerner IM (1950). Heritability of threshold characters. Genetics.

[45] Falconer DS (1965). The inheritance of liability to certain diseases, estimated from the incidence among relatives. Ann. Hum. Genet..

[46] Davey Smith G (2019). Post-modern epidemiology: when methods meet matter. Am. J. Epidemiol..

[47] Davey Smith G (2012). Epigenesis for epidemiologists: does evo-devo have implications for population health research and practice?. Int. J. Epidemiol..

[48] Wang T, Zheng CQ (2005). Roles of cytokines in the pathogenesis of inflammatory bowel disease. World Chin. J. Dig..

[49] Wang W, Sung N, Gilman-Sachs A, Kwak-Kim J (2020). T helper (Th) cell profiles in pregnancy and recurrent pregnancy losses: Th1/Th2/Th9/Th17/Th22/Tfh cells. Front. Immunol..

[50] Saito S, Nakashima A, Shima T, Ito M (2010). Th1/Th2/Th17 and regulatory T-cell paradigm in pregnancy. Am. J. Reprod. Immunol..

[51] Lim KJH (2000). The role of T-helper cytokines in human reproduction. Fertil. Steril..

[52] Bakheet SA (2017). Resveratrol ameliorates dysregulation of Th1, Th2, Th17, and T regulatory cell-related transcription factor signaling in a BTBR T + tf/J mouse model of autism. Mol. Neurobiol..

[53] Abdallah MW (2012). Neonatal levels of cytokines and risk of autism spectrum disorders: an exploratory register-based historic birth cohort study utilizing the Danish Newborn Screening Biobank. J. Neuroimmunol..

[54] Choi GB (2016). The maternal interleukin-17a pathway in mice promotes autism-like phenotypes in offspring. Science.

[55] Fischbach GD, Lord C (2010). The Simons Simplex Collection: a resource for identification of autism genetic risk factors. Neuron.

[56] Daniels AM, Snyder LG, Esler AN, Simon AR (2018). SPARK: A US cohort of 50, 000 families to accelerate autism research NeuroView SPARK: a US cohort of 50, 000 families to accelerate autism research. Neuron.

[57] Kamran Ikram, M. et al. Four novel loci (19q13, 6q24, 12q24, and 5q14) influence the microcirculation In vivo. *PLoS Genet*. **6**, e1001184 (2010). 10.1371/journal.pgen.1001184 PMC2965750 21060863

[58] Kenny L (2016). Which terms should be used to describe autism? Perspectives from the UK autism community. Autism.

[59] Bottema-Beutel K, Kapp SK, Lester JN, Sasson NJ, Hand BN (2021). Avoiding ableist language: Suggestions for autism researchers. Autism in Adulthood.

[60] Chen S, Zhao S, Dalman C, Karlsson H, Gardner R (2021). Association of maternal diabetes with neurodevelopmental disorders: autism spectrum disorders, attention-deficit/hyperactivity disorder and intellectual disability. Int. J. Epidemiol..

[61] Sanders SJ (2015). Insights into autism spectrum disorder genomic architecture and biology from 71 risk loci. Neuron.

[62] Rai D (2013). Parental depression, maternal antidepressant use during pregnancy, and risk of autism spectrum disorders: population based case-control study. Brit. Med. J..

[63] Rai D (2017). Antidepressants during pregnancy and autism in offspring: population based cohort study. Obstet. Gynecol. Surv..

[64] Amiet C (2008). Epilepsy in autism is associated with intellectual disability and gender: evidence from a meta-analysis. Biol. Psychiatry.

[65] Rai D (2018). Association between autism spectrum disorders with or without intellectual disability and depression in young adulthood. JAMA Netw. JAMA Netw. Open.

[66] Altshuler DM (2012). An integrated map of genetic variation from 1,092 human genomes. Nature.

[67] Stergiakouli E (2014). Genome-wide association study of height-adjusted BMI in childhood identifies functional variant in ADCY3. Obesity.

[68] Sun M (2016). Biological insights from 108 schizophrenia-associated genetic loci (METHODS). Nature.

[69] Dudbridge F (2013). Power and predictive accuracy of polygenic risk scores. PLoS Genet..

[70] Jones HJ (2019). Association of Genetic Risk for Rheumatoid Arthritis with Cognitive and Psychiatric Phenotypes Across Childhood and Adolescence. JAMA Netw. Open.

[71] Haycock PC (2016). Best (but oft-forgotten) practices: the design, analysis, and interpretation of Mendelian randomization studies. Am. J. Clin. Nutr..

[72] Gao X (2019). The bidirectional causal relationships of insomnia with five major psychiatric disorders: a Mendelian randomization study. Eur. Psychiatry.

[73] Bowden J, Davey Smith G, Burgess S (2015). Mendelian randomization with invalid instruments: effect estimation and bias detection through Egger regression. Int. J. Epidemiol..

[74] Bowden J, Davey Smith G, Haycock PC, Burgess S (2016). Consistent estimation in Mendelian randomization with some invalid instruments using a weighted median estimator. Genet. Epidemiol..

[75] Hartwig FP, Davey Smith G, Bowden J (2017). Robust inference in summary data Mendelian randomization via the zero modal pleiotropy assumption. Int. J. Epidemiol..

[76] Pedersen CB (2018). The iPSYCH2012 case-cohort sample: new directions for unravelling genetic and environmental architectures of severe mental disorders. Mol. Psychiatry.

[77] Hemani G (2018). The MR-base platform supports systematic causal inference across the human phenome. eLife.

[78] Chen S, Zhao S, Dalman C, Karlsson H, Gardner R (2020). Association of maternal diabetes with neurodevelopmental disorders: autism spectrum disorders, attention-deficit/hyperactivity disorder and intellectual disability. Int. J. Epidemiol..

