# Supplementary material for: Parental inflammatory bowel disease and autism in children
Source: Nat Med. 2022 Jun 2;28(7):1406–11. doi: 10.1038/s41591-022-01845-9 (PMC9307481; doi:10.1038/s41591-022-01845-9)
Supplement: Supplementary file 1 — Supplementary Note. [file 41591_2022_1845_MOESM1_ESM.pdf]

nature portfolio

https://doi.org/10.1038/s41591-022-01845-9

Supplementary information

Parental inflammatory bowel disease and autism in children

In the format provided by the authors and unedited

Parental inflammatory bowel disease and autism in children: Supplementary Information

Supplementary Note: Member list for the iPSYCH Autism Spectrum Disorder working group

Members

Anders D. Børglum [1,2,3]
Jakob Grove [1,2,3,4,5]
Thomas Damm Als [1,2,3]
Thomas Werge [6,7]
Preben Bo Mortensen [1,4,8,9]
Marianne Giørtz Pedersen [8,9,1]
Carsten Bøcker Pedersen [1,8,9,10]
Ole Mors [1,11]
Merete Nordentoft [1,12,6]
David M. Hougaard [1,13]
Jonas Bybjerg-Grauholm [1,13]
Marie Bækvad-Hansen [1,13]
Christine Søholm Hansen [1, 13]

Affiliations

1. The Lundbeck Foundation Initiative for Integrative Psychiatric Research (iPSYCH), Copenhagen, Denmark
2. Department of Biomedicine, Aarhus University, Aarhus, Denmark
3. Center for Genomics and Personalized Medicine (CGPM), Aarhus University, Aarhus, Denmark.
4. Center for Integrative Sequencing (iSEQ), Aarhus University, Aarhus, Denmark.
5. Bioinformatics Research Centre, Aarhus University, Aarhus, Denmark.
6. Department of Clinical Medicine, Faculty of Health and Medical Sciences, University of Copenhagen, Copenhagen, Denmark
7. Institute of Biological Psychiatry, Mental Health Center Sct. Hans, Mental Health Services Copenhagen, Roskilde, Denmark
8. National Centre for Register-Based Research (NCCR), Aarhus University, Aarhus, Denmark
9. Centre for Integrated Register-based Research (CIRRAU), Aarhus University, Aarhus, Denmark
10. Big Data Centre for Environment and Health, Aarhus University, Aarhus, Denmark.
11. Psychosis Research Unit, Aarhus University Hospital, Aarhus, Denmark
12. Copenhagen Mental Health Center, Mental Health Services Capital Region of Denmark Copenhagen, Copenhagen, Denmark
13. Department for Congenital Disorders, Statens Serum Institut, Copenhagen, Denmark
