# Supplementary material for: Parental inflammatory bowel disease and autism in children
Source: Nat Med. 2022 Jun 2;28(7):1406–11. doi: 10.1038/s41591-022-01845-9 (PMC9307481; doi:10.1038/s41591-022-01845-9)
Supplement: Supplementary file 2 — Reporting Summary [file 41591_2022_1845_MOESM2_ESM.pdf]

nature research

Corresponding author(s): Christina Dardani

Last updated by author(s): Apr 20, 2022

Reporting Summary

Nature Research wishes to improve the reproducibility of the work that we publish. This form provides structure for consistency and transparency in reporting. For further information on Nature Research policies, see our Editorial Policies and the Editorial Policy Checklist.

Statistics

For all statistical analyses, confirm that the following items are present in the figure legend, table legend, main text, or Methods section.

n/a	Confirmed	
☐	☒	The exact sample size (*n*) for each experimental group/condition, given as a discrete number and unit of measurement
☐	☒	A statement on whether measurements were taken from distinct samples or whether the same sample was measured repeatedly
☐	☒	The statistical test(s) used AND whether they are one- or two-sided<br>*Only common tests should be described solely by name; describe more complex techniques in the Methods section.*
☐	☒	A description of all covariates tested
☐	☒	A description of any assumptions or corrections, such as tests of normality and adjustment for multiple comparisons
☐	☒	A full description of the statistical parameters including central tendency (e.g. means) or other basic estimates (e.g. regression coefficient) AND variation (e.g. standard deviation) or associated estimates of uncertainty (e.g. confidence intervals)
☐	☒	For null hypothesis testing, the test statistic (e.g. *F*, *t*, *r*) with confidence intervals, effect sizes, degrees of freedom and *P* value noted<br>*Give P values as exact values whenever suitable.*
☒	☐	For Bayesian analysis, information on the choice of priors and Markov chain Monte Carlo settings
☐	☒	For hierarchical and complex designs, identification of the appropriate level for tests and full reporting of outcomes
☐	☒	Estimates of effect sizes (e.g. Cohen's *d*, Pearson's *r*), indicating how they were calculated

Our web collection on statistics for biologists contains articles on many of the points above.

Software and code

Policy information about availability of computer code

Data collection	No software used for data collection.
Data analysis	Data analysis<br>Swedish registry data: STATA/17MP<br>LD score regression: LDSC(LD Score) v1.0.1 software in Python 2.7.18<br>Polygenic risk score estimation: PLINK version 1.9<br>Polygenic risk score analyses: STAT/15MP<br>Two-sample Mendelian randomization: TwoSampleMR R package in R version 3.5.1<br><br>Code availability statement: Analyses were conducted using established protocols for each analytic approach used in the present study. Specifically in the case of LD score regression, the protocol described at: https://github.com/bulik/ldsc/wiki/Heritability-and-Genetic Correlation, was used. In the case of polygenic risk score calculation, the approach described at: https://www.nature.com/articles/nature13595, was applied. Finally, for two-sample Mendelian randomization, the approach described at: https://mrcieu.github.io/TwoSampleMR/articles/introduction.html, was applied.

For manuscripts utilizing custom algorithms or software that are central to the research but not yet described in published literature, software must be made available to editors and reviewers. We strongly encourage code deposition in a community repository (e.g. GitHub). See the Nature Research guidelines for submitting code & software for further information.

Data

Policy information about availability of data

All manuscripts must include a data availability statement. This statement should provide the following information, where applicable:

- Accession codes, unique identifiers, or web links for publicly available datasets
- A list of figures that have associated raw data
- A description of any restrictions on data availability

Swedish registry data: Individual-level data from 'Psychiatry Sweden' were used and under ethics approval from the Stockholm regional ethical review committee (DNR 2010/1185-31/5, 2016/987-32). Due to the sensitive nature of the data, data are not publicly available. Data must remain in the country, according to national laws and registry regulations. Access is restricted to projects approved by the Swedish ethical review authority (https://etikprovningsmyndigheten.se/) and in agreement with the register holders. See https://www.registerforskning.se/en/ for guidance on how to conduct Swedish register-based research. Since there is no central access point for public authority data in Sweden, this process may require coordination with multiple register holders (e.g., Statistics Sweden, The National Board of Health and Welfare) and requires, in our experience, at least one year from the time of ethical approval, depending on workload for each register holder.

GWAS summary data: GWAS summary data for IBD, ulcerative colitis, Crohn's disease and autism used in the LD score regression, polygenic risk score and Mendelian randomization analyses, are publicly available (IBD: http://ftp.ebi.ac.uk/pub/databases/gwas/summary_statistics/GCST004001-GCST005000/GCST004131/; UC: http://ftp.ebi.ac.uk/pub/databases/gwas/summary_statistics/GCST004001-GCST005000/GCST004133/; Crohn's: http://ftp.ebi.ac.uk/pub/databases/gwas/summary_statistics/GCST004001-GCST005000/GCST004132/; Autism: https://www.med.unc.edu/pgc/download-results/). Restrictions apply to the availability of the GWAS summary data for autism without intellectual disabilities, in order to ensure that there is no conflict with ongoing projects, collaborations and iPSYCH's data sharing policies. Data can accessed after correspondence with the iPSYCH: https://ipsych.dk/. Researchers will be asked to prepare a short application briefly describing the proposed study and responses will typically be within 2 weeks.
1000 Genomes project European data used in LD score regression is publicly available (from: https://data.broadinstitute.org/alkesgroup/LDSCORE/eur w ld_chr.tar.bz).

ALSPAC data: Ethical approval for the study was obtained from the ALSPAC Ethics and Law Committee and the Local Research Ethics Committees. Individual-level data from the ALSPAC birth cohort are not publicly available for reasons of clinical confidentiality. Data can be accessed after application to the ALSPAC Executive Team who will respond within 10 working days. Application instructions and data use agreements are available at http://www.bristol.ac.uk/alspac/researchers/access/.

Field-specific reporting

Please select the one below that is the best fit for your research. If you are not sure, read the appropriate sections before making your selection.

☒ Life sciences ☐ Behavioural & social sciences ☐ Ecological, evolutionary & environmental sciences

For a reference copy of the document with all sections, see nature.com/documents/nr-reporting-summary-flat.pdf

Life sciences study design

All studies must disclose on these points even when the disclosure is negative.

Sample size

The Swedish registry cohort study was conducted in a sample of 2,324,227 offspring born to 1,282,494 mothers and 1,285,719 fathers. Post hoc power calculations suggested that we had over 80% power to detect an odds ratio of 1.1.
For the LD score, polygenic risk score estimation and two-sample Mendelian randomization studies we used the latest publicly available summary data, to increase the power of the analyses. Specifically: autism (Ncases= 18,381; Ncontrols= 27,969), autism excluding intellectual disabilities cases (Ncases= 11,203; Ncontrols= 22,555), IBD (Ncases= 25,042; Ncontrols= 34,915), Crohn's (Ncases= 12,194; Ncontrols= 28,072) and UC (Ncases= 12,366; Ncontrols= 33,609).
Finally, for the polygenic risk score analyses in the ALSPAC birth cohort, we used as an outcome variable the autism factor mean score, a broad autism phenotype measure available in ~13,000 children in the cohort. This ensured we would have the largest sample size possible for these analyses in ALSPAC.

Data exclusions

Case definition and participant exclusion criteria for the Swedish registry cohort study are illustrated in Figure S1 of the supplementary material. In summary, exclusion criteria were: children born outside Sweden (n=292,023), not registered in the Medical Birth Register (n=74,240), resident in Sweden for <5 years (n=23,495), multiple pregnancy (n=67,309), adopted (n=2,425), known genetic/metabolic causes of neurodevelopmental conditions (e.g. trisomies) (n=7,873) or incomplete parental records (n=45,453).

In the case of LD score regression, polygenic risk score analyses and two-sample Mendelian randomization, all available data were used after applying quality control procedures suggested from the developers of the approaches (https://github.com/bulik/ldsc/wiki/Heritability-and Genetic-Correlation; https://www.nature.com/articles/nature13595; https://mrcieu.github.io/TwoSampleMR/articles/introduction.html).
Quality control procedures for the ALSPAC genotype data can be found in Supplementary Methods S2 of the supplement and in Stergiakouli E, Gaillard R, Tavaré JM, et al. Genome-wide association study of height-adjusted BMI in childhood identifies functional variant in ADCY3. Obesity (Silver Spring). 2014;22(10):2252-2259.

In LD-score regression quality control steps, single nucleotide polymorphisms (SNPs) are excluded if they are not present in the 1000 Genomes European LD-reference panel, they have missing values, imputation INFO scores <= 0.9, minor allele frequency <=0.01, have out-of-bounds p-values, are strand-ambiguous or duplicates.

In PRS quality control Individuals were excluded on the basis of the following filters:
1. gender mismatches,
2. undetermined X chromosome heterozygosity,
3. over 3% missingness (children), over 5% missingness (mothers),
4. evidence of crypted relatedness; >10% of shared alleles identical by descent in children and >12.5% of shared alleles identical by descent in mothers,
5. non-European ancestry, assessed by multidimensional scaling analysis compared to HapMap 2 individuals.
SNPs were excluded on the basis of the following filters:
1. minor allele frequency <1%,
2. call rate < 95%,
3. Hardy-Weinberg equilibrium (HWE) p<5e-07.
Also SNPs with mismatching alleles between the discovery and target dataset were removed. The MHC region was removed (25 Mb – 34 Mb), except for one SNP representing the strongest signal within the region.

For Mendelian randomization, SNPs were excluded if they were not present in both the exposure and outcome GWAS. During the harmonization step, SNPs were excluded if they were palindromic (i.e. the alleles on the forward strand are the same as the reverse strand) as this prevented correct harmonization.

Replication

We used four complementary designs. We found evidence of associations between maternal diagnosis and polygenic risk for IBD subtypes and offspring autism. This was further supported by two-sample Mendelian randomization analyses suggesting a causal effect of genetic liability to ulcerative colitis on autism. Despite triangulating our findings, replication in other international cohorts is necessary to build on the findings and their possible explanations. No direct replication of each individual analysis was done.

Randomization

In the Swedish registry cohort study we controlled for covariates including: parental age at delivery, migrant status, education level, family income quintile at birth, parents' history of psychiatric diagnosis prior to the birth of the child and offspring sex, birth year and birth order. We additionally mutually adjusted for maternal and paternal IBD diagnoses to avoid bias from assortative mating.
In the ALSPAC polygenic risk score analysis we controlled for child's sex and the first 10 principal components of the ALSPAC genotype data to avoid population stratification bias. Finally, Mendelian randomization has been compared to a natural randomized controlled trial, as genetic variants used as instruments are allocated randomly during meiosis. This is similar to blinding at allocation.

Blinding

Blinding of the samples was not applied and not relevant as no interventions were conducted.

Reporting for specific materials, systems and methods

We require information from authors about some types of materials, experimental systems and methods used in many studies. Here, indicate whether each material, system or method listed is relevant to your study. If you are not sure if a list item applies to your research, read the appropriate section before selecting a response.

Materials & experimental systems

n/a	Involved in the study
☒	☐ Antibodies
☒	☐ Eukaryotic cell lines
☒	☐ Palaeontology and archaeology
☒	☐ Animals and other organisms
☒	☐ Human research participants
☒	☐ Clinical data
☒	☐ Dual use research of concern

Methods

n/a	Involved in the study
☒	☐ ChIP-seq
☒	☐ Flow cytometry
☒	☐ MRI-based neuroimaging
